# Chitinase family GH18: evolutionary insights from the genomic history of a diverse protein family

**DOI:** 10.1186/1471-2148-7-96

**Published:** 2007-06-26

**Authors:** Jane D Funkhouser, Nathan N Aronson

**Affiliations:** 1 Department of Biochemistry and Molecular Biology, College of Medicine, University of South Alabama, Mobile, Alabama 36688, USA

## Abstract

**Background:**

Chitinases (EC.3.2.1.14) hydrolyze the β-1,4-linkages in chitin, an abundant N-acetyl-β-D-glucosamine polysaccharide that is a structural component of protective biological matrices such as insect exoskeletons and fungal cell walls. The glycoside hydrolase 18 (GH18) family of chitinases is an ancient gene family widely expressed in archea, prokaryotes and eukaryotes. Mammals are not known to synthesize chitin or metabolize it as a nutrient, yet the human genome encodes eight GH18 family members. Some GH18 proteins lack an essential catalytic glutamic acid and are likely to act as lectins rather than as enzymes. This study used comparative genomic analysis to address the evolutionary history of the GH18 multiprotein family, from early eukaryotes to mammals, in an effort to understand the forces that shaped the human genome content of chitinase related proteins.

**Results:**

Gene duplication and loss according to a birth-and-death model of evolution is a feature of the evolutionary history of the GH18 family. The current human family likely originated from ancient genes present at the time of the bilaterian expansion (approx. 550 mya). The family expanded in the chitinous protostomes *C. elegans *and *D. melanogaster*, declined in early deuterostomes as chitin synthesis disappeared, and expanded again in late deuterostomes with a significant increase in gene number after the avian/mammalian split.

**Conclusion:**

This comprehensive genomic study of animal GH18 proteins reveals three major phylogenetic groups in the family: chitobiases, chitinases/chitolectins, and stabilin-1 interacting chitolectins. Only the chitinase/chitolectin group is associated with expansion in late deuterostomes. Finding that the human GH18 gene family is closely linked to the human major histocompatibility complex paralogon on chromosome 1, together with the recent association of GH18 chitinase activity with Th2 cell inflammation, suggests that its late expansion could be related to an emerging interface of innate and adaptive immunity during early vertebrate history.

## Background

The recent increase in genome projects has provided DNA sequence data useful for understanding the evolutionary dynamics that resulted in the conservation of ancient proteins as modern protein families. One such family of proteins is the glycoside hydrolase family 18 chitinases (GH18), widely expressed in archea, prokaryotes and eukaryotes. Definition of this family is based on amino acid sequence similarity [1-3]. In eukaryotes the proteins are mainly expressed by fungi, arthropods and nematodes (for review see [4]), but they are also expressed in mammals, with members recently shown to be involved in important physiological processes that include T-cell mediated inflammation and allergy [5,6]. The chitinases hydrolyze chitin, an abundantly produced homopolymer of N-acetyl-β-D-glucosamine that provides architectural reinforcement of biological structures such as insect exoskeletons, fungal cell walls, nematode egg shells, and other biological matrices involved in protection and self defense. Chitin has not been detected in higher plants and vertebrates, where cellulose and hyaluronan, respectively, may replace certain chitin functions [7,8]. In chitinous species, the chitinases along with chitin synthetases are essential for remodeling chitin-containing structures during growth and development. Certain bacterial genera and plants, which do not produce chitin, use chitinases, respectively, for the conversion of insoluble chitin into metabolizable nutrient sources and for defense from chitinous pathogens.

Mammals are not known to synthesize chitin or to metabolize it as a nutrient, yet the human genome encodes eight well-documented genes for proteins now classified as glycoside hydrolase family18 members. Members of this family are known to adopt the TIM (triosephosphate isomerase) fold consisting of a strongly conserved (β/α)_8_-barrel structure [1-3]. Often, separate chitin-binding domains (CBM14) [2,3] are present in the carboxyl terminal region of the proteins (additional file 1: GH18 family domain structure). The protein family includes chitinases as well as homologous proteins termed chitolectins. The latter lack the key active-site glutamate residue that donates a proton required for hydrolytic enzyme activity, but retain highly conserved residues involved in oligosaccharide binding and overall three-dimensional structure. Traditionally, chitinases are classified in two glycoside hydrolase families, GH18 and GH19, with different structures and catalytic mechanisms [9]. Family GH18 includes the chitinases from viruses, bacteria, fungi and animals as well as classes III and V from plants [10]. The GH19 chitinases are identified mostly in plants (classes I, II and IV), nematodes, and some bacteria [11]. Recent data indicate chitinase activity is also present in protein families GH48 and GH20 [12,13]. N-acetyl-β-D-glucosaminidases such as those in family GH20 also can participate in chitin degradation by hydrolyzing GlcNAc from the non-reducing end of chito-oligosaccharides [14].

Three of the GH18 proteins encoded by the human genome have demonstrated enzymatic activity. They are: di-N-acetylchitobiase, active in the lysosomal degradation of asparagine-linked glycoproteins [15]; chitotriosidase, a vesicular and secreted protein produced by activated macrophages and highly elevated in serum from patients with Gaucher disease [16]; and acidic mammalian chitinase (AMCase), expressed mostly in the gastrointestinal tract and lung [17], and recently shown to be induced by Th2-helper cells in an aeroallergen model of asthma [5]. A gene similar to the AMCase gene encodes a fourth hypothetical protein with a truncated GH18 domain. Three human genes (OVGP1, CHI3L1 and CHI3L2) encode chitolectins likely to be involved in tissue remodeling during inflammation and/or development. The OVGP1 gene encodes a large glycoprotein secreted by oviduct epithelial cells in response to estrogen [18,19]. The glycoprotein includes an extended C-terminal repeat region similar to sequences found in mucins. A number of cell types, including macrophages, articular chrondrocytes and synovial cells, secrete proteins encoded by the CHI3L1 and CHI3L2 genes under inflammatory conditions [20-22]. A recently identified CHID1 gene encodes a lysosomal GH18 chitolectin that interacts with stabilin-1, an endocytic/sorting receptor of macrophages that serves as a marker of alternative activation [23]. The function of the CHID1 protein is not yet established, and the sequence similarity to other GH18 proteins is low.

Although the biological functions of most human GH18 proteins are not yet delineated, the available protein characterizations indicate that a process of gene duplication and diversification has resulted in multiple functions that are not related to nutrient utilization or growth-related turnover of chitinous structures. Instead, recent data pertaining to the mammalian proteins point to their prominent roles in defense against fungal or other pathogens and in inflammation and remodeling processes [6]. In this communication, we addressed the evolutionary history of the GH18 multiprotein family from early eukaryotes to mammals in an effort to understand the forces that shaped the human genome content of chitinase-related proteins. The present study indicates that over evolutionary time the GH18 family evolved by decline and expansion according to selective forces associated with speciation. An expansion of chitinase genes occurred as chitinous species appeared early in metazoan evolution, a decline followed as chitin disappeared as an important structural and protective feature of early deuterostomes, and a second expansion began in non-chitinous vertebrates where chitinases and chitolectins may have evolved to assume increasingly important roles associated with pathogen recognition, processing and antigen presentation.

## Results

### Human GH18 Family Genes and Their Relationship to Other Mammalian GH18 Genes

Seven of the eight human GH18 family members are located on chromosome 1. The chitobiase gene is located at 1p22, 26 Mb upstream of the regions containing six other chitinase/chitolectin genes, which cluster into two groups, at 1p13 and at 1q32 (Table 1): Genes CHI3L2, CHIA, RP11-165H20.1 and OVGP1 cluster at 1p13; and CHIT1 and CHI3L1 at 1q32. Only the recently annotated and weakly homologous CHID1 gene is not located on chromosome 1, but at chromosome 11p15.5. A similar but unique pattern of homologues is present in the mouse genome, which includes ten genes encoding GH18 family members. The genes are located on mouse chromosomes 1, 3 and 7. Genes Ctbs, Chia, Chi3l3, Chi3l4, Ovgp1, Bclp2 and BC051070 are located on chromosome 3, and Chit1 and Chi3l1 are on chromosome 1. The mouse CHID1 homolog, designated 3110023E09Rik [Entrez Gene ID 68038], is located at chromosome 7F5. The rat genome is not complete, but homologous rat genes are located on chromosomes 2, 13 and 18. There is conservation of synteny in the chromosomes of these three mammals that contain GH18 family members.

**Table 1:** Human Genes Encoding GH18 Family Members

Entrez Gene ID	Gene (Aliases)	Chr^a ^Location	Protein Accession	Description
27159	CHIA	1p13.2	NP_970615 & NP_068569	acidic chitinase (AMCase, eosinophil chemotactic cytokine)
1118	CHIT1	1q32.1	NP_003456	chitinase 1 (chitotriosidase)
1116	CHI3L1 (YKL 40, GP39, TSA1902)	1q32.1	NP_001267	chitinase 3-like 1 (cartilage protein 39)
1117	CHI3L2 (YKL 39)	1p13.3	NP_003991	chitinase 3-like 2 (chondrocyte protein 39)
RP11-165H20.1	(LOC149620)	1p13.2	NP_001013643	NCBI Ref Seq: NP_001013643 was permanently suppressed because it is a nonsense mediated mRNA decay (NMC) candidate
5016	OVGP1	1p13.2	NP_002548	oviductal glycoprotein 1; oviductin
1486	CTBS	1p22	NP_004379	chitobiase, di-N-acetyl
66005	CHID1	11p15.5	NP_076436	stabilin-1 interacting chitinase-like protein

^a ^Chromosome

**Figure 1:**
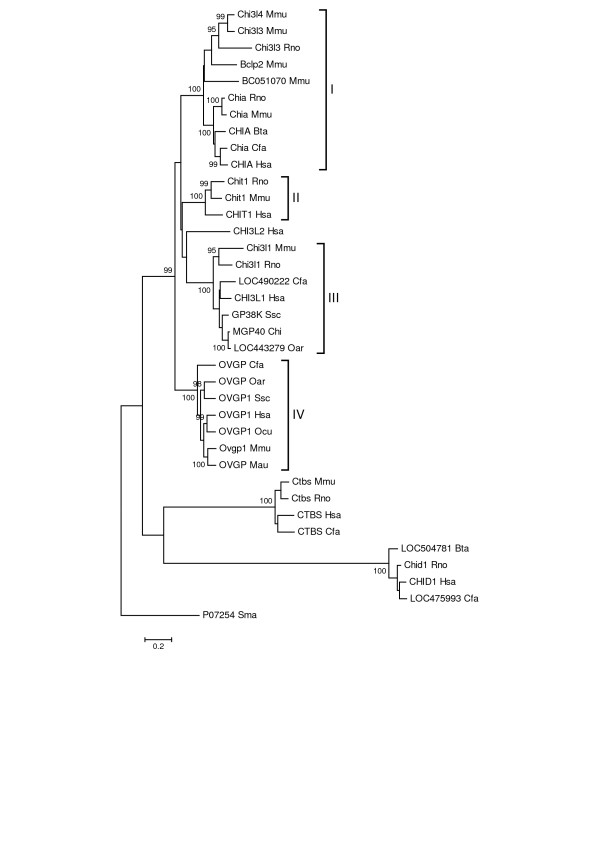
**Phylogenetic tree of GH18 domains from 36 mammalian proteins**. The phylogenetic trees presented in this paper were constructed with Mega version 3.1 software [86]. Minimum evolution, neighbor-joining, and maximum parsimony methods were used with Poisson correction for multiple amino acid substitution and with 1000 random bootstrap replicates. The figure shows the minimum evolution tree (the three methods produced very similar topologies). Alignment of the GH18 domain amino acid sequences used ClustalX (1.83) [84]. Gene symbols (or protein symbols where genes symbols are not available) and binomen species abbreviations of the form "Gsp" (Genus, species) are used. The GH18 proteins comprise three major phylogenetic groups, with the chitinase/chitolectin group forming four subgroups denoted I, II, III and IV (discussed in the text). The scale at the bottom left is in units of amino acid substitutions per site. Bootstrap values ≥ 95% are shown. The tree is rooted with the *Serratia marcescens *family GH18 chitinase A [GenBank:P07254].

From the genome sequences currently available, it is evident based on protein sequence similarities that the mammalian genomes include orthologous clades of GH18 family members, and that the chitinase/chitolectin clade (Figure 1) includes orthologous groups of paralogous family members. Construction of a phylogenetic tree from alignment of GH18 domains from 36 mammalian proteins (additional file 2: Mammalian Sequences) illustrates these relationships. Figure 1 shows the tree, constructed using the minimum evolution method. The lysosomal chitobiases and stabilin-1 interacting chitolectins form outlying clades of orthologous proteins distinct from the true chitinases and chitolectins, which form four separate subgroups. Subgroup I represents the acidic chitinases and related chitolectins; subgroup II, the chitotriosidase enzymes; subgroup III, the non-enzymatic proteins associated with injury, repair and remodeling; and subgroup IV, the oviduct glycoproteins and related sequences. Each of the groups is not represented in all the genomes. This may be explained by genome projects that are not complete; or gene duplication or gene loss may have occurred relatively late in vertebrate evolution. Among GH18 enzymes, chitobiase is biochemically unique as the only member that splits off a monosaccharide from the reducing end of chito-oligosaccharides [15].

### Chitinase/Chitolectin Genes in Early Eukaryotes (*D. discoideum *and *H. echinata*)

**Figure 2:**
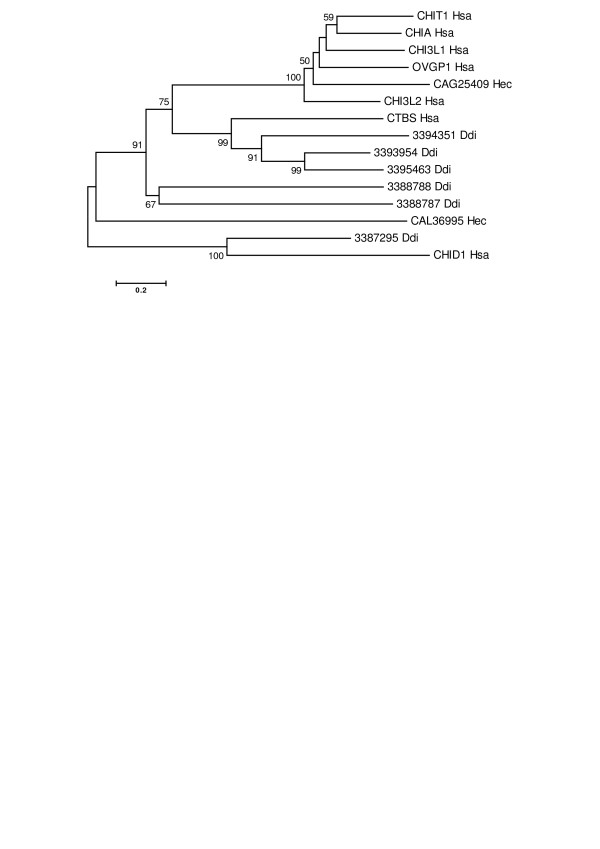
**Phylogenetic relationship of *D. discoideum, H. echinata*, and human GH18 proteins**. The figure shows a minimum evolution tree from a phylogenetic analysis conducted as described for Figure 1. Gene (or protein symbols when gene symbols are not available) and binomen species abbreviations are used. The tree is rooted at midpoint. Bootstrap values ≥ 50% are shown.

To assess the family GH18 genes in early eukaryotes and their relationship to the human genes, we examined the genome of the social amoeba *Dictyostelium discoideum*. *D. discoideum *diverged from the animal-fungal lineage after the plant-animal split, and has retained a significant amount of the diversity of the ancestral eukaryotic genome [24]. Thus, chitinases present in the *D. discoideum *genome should provide evolutionary insight into the ancestral metazoan GH18 proteins present before an apparent expansion of chitinases associated with the biological importance of chitin in protostomes (discussed in the next section). A search of the *D. discoideum *genome database at NCBI (Build 1.1;November 22, 2005) revealed six genes encoding chitinase-related proteins. Five of the genes cluster on two chromosomes: a 2.6 Mbp region of chromosome 2 encodes Entrez genes 3393954, 3395463, and 3394351; and a 3.5 Kb region of chromosome 5 encodes Entrez genes 3388787 and 3388788. A sixth gene (Entrez Gene 3387295) also located on chromosome 5 encodes a hypothetical protein [GenBank:XP_635730] that aligned with the human stabilin-1 interacting protein (Expect = 2e-48). Phylogenetic analysis (Figure 2) indicated that the three chromosome 2 genes are co-orthologs of the human chitobiase. Two chromosome 5 genes are more distant homologs, distinct from the human chitinases/chitolectins. The third chromosome 5 gene is a human CHID1 ortholog. If the eukaryotic ancestral genome included orthologs of the mammalian chitinase/chitolectin group, and the presence of chitinases in plants [11] indicates it did, they likely have been lost in the *D. discoideum *lineage. As the cells of *Dictostelium *are professional phagocytes that consume bacteria as a primary nutritional source [25], the chitobiases and the related proteins are likely involved in the uptake, internalization, killing and subsequent digestion of bacterial cell wall components. Indeed, chitobiase will hydrolyze GlcNAcβ-D-(1–4)MurNAc, the repeating disaccharide unit of the bacterial cell wall peptidoglycan [26]. However, the proteins also may contribute to the control of microbial growth within the digestive phagosomes [27].

Further information about the early evolution of chitin-degrading proteins comes from the recent cloning of a cDNA encoding a chitinase from a chitinous basal metazoan, the cnidarian *Hydractinia echinata *[28]. The 1467 bp cDNA encodes a typical GH18 glycoside hydrolase of 464 amino acids [GenBank:CAG25409]. The protein sequence includes a secretory signal at the N-terminus and a highly conserved catalytic motif including its hallmark proton-donating glutamic acid. The highest scoring human sequence aligned by NCBI BLAST with the *H. echinata *protein sequence (Expect = 3e-90) is chitotriosidase [GenBank:NP_003456] (44% identity and 59% similarity over 414 residues, including the GH18 domain). However, all the human chitinases/chitolectins produced high scoring alignments (Expect < 2e-57). In contrast to the proteins from *D. discoideum*, the phylogenetic analysis (Figure 2) indicated orthology of the CAG25409 protein with the human chitinases/chitolectins.

Although *H. echinata *feeds on small crustaceans, other unidentified chitinase(s) may participate in their digestion [28]. (A second more recently identified *H. echinata *protein [NCBI accession CAL36995] with annotated glycosyl-hydrolase and chitin-binding domains is not closely related to the human or *Dictyostelium *proteins, Figure 2; but NCBI BLAST identifies high scoring alignments with fungal chitinases.) The cDNA expression patterns determined by in situ hybridization indicated that the CAG25409 protein has a role in body pattern formation and immunity. *H. echinata *proteins involved in immunity are of particular interest because *H. echinata *has well developed allo-recognition responses that may relate to the early evolution of vertebrate and nonvertebrate immune systems [29]. Thus, functions of this early metazoan chitinase suggest an ancestral pattern of chitinase/chitolectin involvement in differentiation, development and immunity that continued from the earliest metazoans to present mammals. The growth and developmental function is evident in imaginal disc growth factors and chitinase-like proteins from the protostomes *D. melanogaster *and *C. gigas *(see next section) and in human chitolectins including OVGP1, CHI3L1, and CHI3L2, which are secreted proteins reported to be involved in recognition processes associated with fertilization, tissue inflammation, and wound repair [19,30-33].

### Evolutionary Relationship between Chitinase-Related Genes from Human and Protostomes (*D. melanogaster *and *C. elegans*)

As expected, comparison of family GH18 genes from genera of animals from which genome sequences are complete indicated that the highly chitin-metabolizing protostomes *C. elegans *and *D. melanogaster *have more chitinase-related genes. A search of the *C. elegans *database at the NCBI and other resources such as Wormbase [34] and ACEView [35] identified 37 genes or predicted genes encoding chitinases and related GH18 family members (additional file 3: *C. elegans *Sequences). Searches of *D. melanogaster *genome resources at NCBI and FlyBase [36] identified 17 recognized or predicted genes encoding GH18 family proteins (additional file 4: *D. melanogaster *Sequences). Many of these protostome genes were identified in earlier publications [37,38].

**Figure 3:**
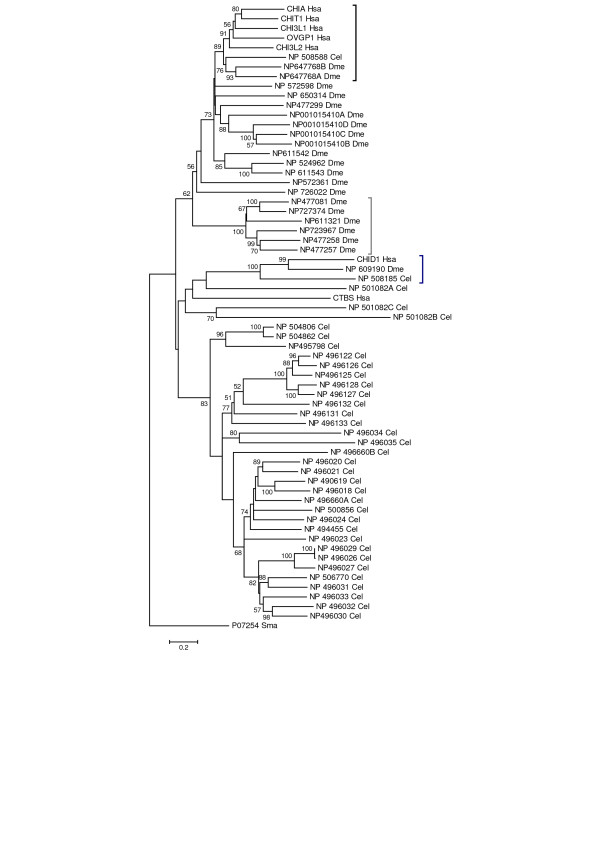
**Phylogenetic analysis of GH18 domains of chitinase/chitolectins from human, *D. melanogaster *and *C. elegans***. The figure shows a minimum evolution tree from a phylogenetic analysis conducted as described in the legend for Figure 1. Binomen abbreviations are used for species, gene symbols for human proteins, and accession numbers for *D. melanogaster *and *C. elegans *proteins. A black bracket at the right indicates the clade encompassing all the human chitinases/chitolectins, two domains from a single protein of *D. melanogaster*, and a single protein of *C. elegans*; a dark gray bracket indicates the clade encompassing the *D. melanogaster *imaginal disc growth factors (chitolectins); and a dark blue bracket the stabilin-1 interacting chitolectin group. Scale, bootstrapping and rooting are as described for Figure 1. Bootstrap values ≥ 50% are shown.

A phylogenetic tree created from multiple alignment of GH18 domain sequences from *C. elegans*, *D. melanogaster *and human proteins indicated a general lack of orthology among the members of the families of GH18 proteins (Figure 3). The human chitinases/chitolectins grouped with high significance with only a single protein from each protostome. In the case of *C. elegans*, all the human proteins grouped with the worm chitinase, cht-1 [GenBank:NP_508588], encoded on the X chromosome. The gene encodes a predicted 66.9 kD protein with one GH18 domain and two N-terminal CBM14 (chitin binding) domains. This domain structure differs from the vertebrate enzymes, where a single CBM14 domain is present in each of the enzymatically active chitinases (CHIT1 and AMCase). The *C. elegans *cht-1 protein aligned with the human CHIT1 protein with 40% identity and 56% similarity over 477 residues, including the GH18 domain and a CBM14 domain. For *D. melanogaster*, domains from a single protein [GenBank: NP_647768] located on chromosome 3L at 3.07 Mb, clustered with the human GH18 domains. The NP_647768 protein comprises two GH18 domains but does not include a CBM14 domain. The two domains aligned with the human CHIT1 protein over 367–375 residues with 45–52% identity and 65% similarity. The results from the phylogenetic analysis of human and protostomes sequences (Figure 3) indicate a single clade encompasses these two proteostome proteins and all the human GH18 family proteins except the two outlying groups (Figure 1). The separation of this clade (bootstrap value 89%) suggests that the human GH18 chitinases/chitolectins are likely co-orthologs of the cht-1 protein from *C. elegans *and the NP_647768 protein from *D. melanogaster*. Interestingly, an analysis that included the *H. echinata *chitinase placed it in the same clade (bootstrap value 85%) (additional file 6: Supplemental phylogenetic tree). From these data, we infer that the present day *H. echinata *CAG25409 protein, the *C. elegans *NP_508588 protein, and the *D. melanogaster *NP_647768 protein are descended from an ancient common ancestor; and the human chitinases/chitolectins appear to be lineal descendents of this ancestral protein.

Further, the phylogenetic analysis of the protostome sequences indicated that *D. melanogaster *and *C. elegans *genomes each included a one-to-one ortholog for the human stabilin-1 interacting chitolectin. The GH18 domains from the *C. elegans *protein [GenBank:NP_508185], the *D. melanogaster *protein [GenBank:NP_609190], and the human CHID1 gene product formed a monophylogenic group supported by a bootstrap value of 100% (Figure 3). In contrast, sequence alignments and phylogenetic analyses did not identify unequivocal protostome orthologs for the human chitobiase (CTBS).

So far, the *C. elegans *GH18 family members are only predicted from sequence relationships, and their physiological functions are not yet documented. Many of the *C. elegans *proteins include only the GH18 domain, but some sequence annotations include conserved domains known to bind chitin, oligosaccharides and peptidoglycans. The functions of some of the *D. melanogaster *proteins, however, are well established. They include enzymatic functions involved in chitin turnover associated with molting [4] as well as a more recently characterized cell proliferation role that promotes growth in imaginal discs [39,40]. These growth-promoting factors (IDGFs) comprise a family of five soluble proteins. They differ from the chitinases in that they lack the C-terminal chitin-binding domain, and glutamine replaces the critical active-site glutamate residue involved in the catalytic mechanism of all known GH18 chitinase enzymes. The GenBank accession numbers for the IDGFs are NP_477258, NP_477257, NP_723967, NP_727374 and NP_611321. They form a monophyletic group supported by a bootstrap value of 100% (Figure 3). One protein [GenBank:NP_477081] included in the group is not annotated as IDGF; however, BLASTP identifies its alignments with IDGFs as the best hits in the *D. melanogaster *genome. Interestingly, although some mammalian chitolectins of subgroups I, III and IV (Figure 1) are associated with growth, differentiation and remodeling, they did not group with the imaginal disc growth factors in the phylogenetic analysis (Figure 3). In contrast to the polar glutamine in *D. melanogaster *imaginal disc factors, the substitutions for the catalytic glutamate residue in the mammalian subgroups III and IV proteins OVGP1, CHI3L1 and CHI3L2 are the non-polar residues leucine or isoleucine. The mouse Chi3l3 (Ym1) and Chi3l4 (Ym2) proteins of subgroup I (Figure 1), however, have glutamines substituted for the active-site glutamates.

The three-dimensional structures of some of the chitolectins, including IDGF2, are available [40-42]. Analysis of the structure-function properties of IDGF2 suggests the interesting possibility that the *D. melanogaster *IDGFs may have evolved to acquire new functions as growth factors by an evolutionary progression from carbohydrate-binding to protein-binding molecules [40]. The 3D structures of the binding sites of two of the mammalian chitolectins, human cartilage glycoprotein-39 (CHI3L1) [GenBank:NP_001267] and mouse Ym1 (Chi3l3) [GenBank:NP_034022], support carbohydrate binding in a manner similar to that observed for active chitinases, with no indication of the evolutionary progression to protein binding as proposed for IDGF2 [41,42]. This is consistent with our phylogenetic analysis, which shows a much closer evolutionary relationship of the human chitolectin proteins to a putative chitinase [GenBank:NP_647768] and several other *D. melanogaster *proteins than to the IDGFs (Figure 3). However, based on a recent higher resolution structure of the mouse Ym1 protein, the carbohydrate-binding groove in this mammalian chitolectin may be somewhat different from that of the human cartilage glycoprotein-39 (CHI3L1) [43].

Recently two family GH18 proteins were cloned from the oyster *Crassostrea gigas*, providing the first information about the protein family from the Lophotrochozoan branch of protostomes [44,45]. The proteins were *Cg*-Clp1 [GenBank:CAI96028], a chitinase-like protein homologous to the human CHI3L1 (GP-39), and *Cg*-Chit [GenBank:CAI96026], an atypical family 18 chitinase. Data from gene expression profiles and biochemical characterization indicated the *Cg*-Clp1 protein is involved in control of growth and development, as reported for the *D. melanogaster *IDGF proteins. The *Cg*-Chit protein characterization, however, suggests that it plays an important role as an immunity effector in molluscs. Analysis of phylogenetic relationships of the *Cg*-Clp1 protein with proteins from other branches of Bilateria supported a closer relationship of the *Cg*-Clp-1 protein to mammalian chitinases/chitolectins than to the *D. melanogaster *IDGFs [44]. Currently, six GH18 domain protein sequences from *Crassostrea gigas *are available from the NCBI protein database [GenBank: CAI96025, CAI96026, CAI96027, CAI96028, CAI96023, CAI96024]. Three of the sequences, including *Cg*-Chit (Chit3) [GenBank:CAI96026], include the proton-donating glutamate residue in the catalytic motif, indicating that they are active chitinases. The other three sequences have a glutamine residue substituted for the glutamate, as seen in *D. melanogaster *IDGF proteins, and are likely chitolectins. We created phylogenetic trees from sequence alignments of the oyster GH18 domains with domains from *C. elegans*, *D. melanogaster*, and from selected mammalian species and, as reported for the *Cg*-Clp1 protein [44,45], found support for an ancestral relationship of *C. gigas *proteins with the mammalian chitinase/chitolectins. Our analysis supported separate monophylogenic groups for the human chitolectins, *D. melanogaster *IDGF proteins, and the *C. gigas *chitinase-like proteins.

The phylogenetic relationships between the human chitinase/chitolectin sequences and the *C. elegans *and *D. melanogaster *sequences shown in Figure 3 suggest that the present multigene chitinase/chitolectin families in each individual species reflect unique innovations that accompanied the evolutionary radiation of bilaterians into the protostome and deuterostome divisions. The limited orthology of the vertebrate chitinase/chitolectin family with the protostome chitinases/chitolectins is consistent in both deuterostomes and protostomes with extensive gene duplication, followed by retention and diversification as well as gene loss after the radiation. In *C. elegans *and *D. melanogaster*, expansion and diversification of chitinases likely were driven by the appearance and importance of chitinous structures in nematodes and insects and the need for proteins associated with chitin turnover in development, protection and metabolism. The factors important in the human genome, however, are more difficult to understand. Clearly, the human chitinase/chitolectin proteins are more similar to each other than to homologs in *C. elegans *and *D. melanogaster*. On this basis, we infer that most of the human genes are relatively recent products of gene duplication events. The genes produced by the duplications therefore have evolved in vertebrates in the absence of chitin biosynthesis.

### Chitinases/Chitolectins in Early Deuterostomes (*C. intestinalis *and *S. purpuratus*)

The expansion and diversification of chitinase-related genes observed in the protostomes *C. elegans *and *D. melanogaster *apparently did not occur in the earliest deuterostomes. Available data from the urochordate *Ciona intestinalis *(sea squirt) draft genome as well as from echinodermata *Strongylocentrotus purpuratus *(sea urchin) indicate far fewer chitinase-related genes in non-chitinous organisms than already observed for *C. elegans *and *D. melanogaster*. The availability of these genomes provides an opportunity to examine chitinase-related genes from genomes highly relevant for elucidation of the ancestral state prior to two rounds of genome duplication postulated to have occurred in a lineage leading to vertebrates [46-48].

**Figure 4:**
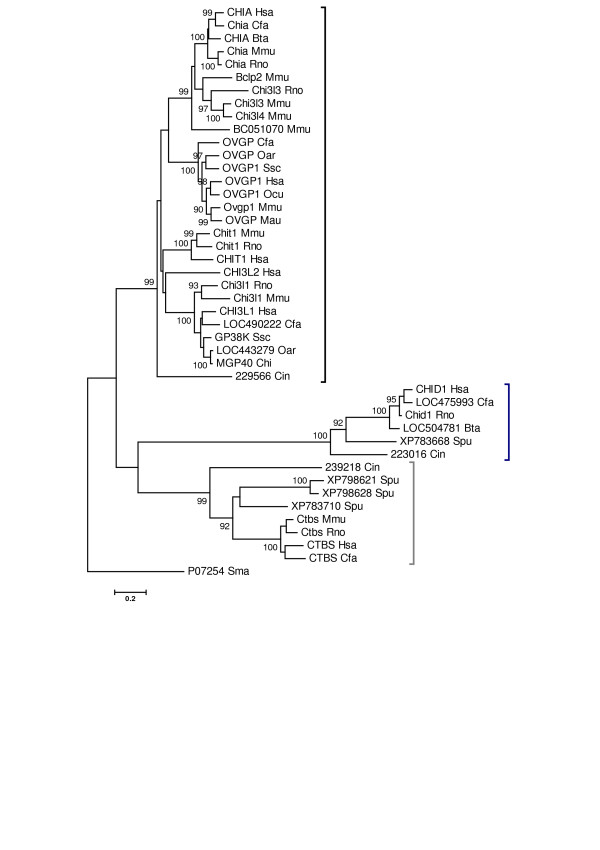
**Phylogenetic analysis of GH18 family members from mammals, *C. intestinalis *and *S. purpuratus***. The figure shows the minimum evolution tree from a phylogenetic analysis conducted as described in the legend for Figure 1. JGI model protein ID numbers are used for *C. intestinalis *proteins, accession numbers for the *S. purpuratus *proteins, and gene symbols (or protein symbols when gene symbols are not available) for mammalian proteins. A black bracket indicates the chitinases/chitolectins, a dark blue bracket the stabilin-1 interacting chitolectins, and a dark gray bracket the chitobiases.

Searches of the data available from the JGI (Joint Genome Institute) *C. intestinalis *draft genome v2.0 (March 2005) [49] and from *S. purpuratus *(genome assembly build 1.1; August 2005) identified three sequences from the *C. intestinalis *genome and four from the *S. purpuratus *genome that produced high scoring alignments with human GH18 family members (Accession numbers listed in additional file 5: Other deuterostome sequences). Figure 4 shows a phylogenetic tree created from an alignment of the *C. intestinalis *and *S. purpuratus *sequences with mammalian GH18 protein sequences. The analysis indicates that the mammalian chitinases/chitolectins are co-orthologs of *C. intestinalis *JGI gene model protein ID229566. The mammalian chitobiases and stabilin-1 interacting proteins are orthologs, respectively, of the other two *C. intestinalis *proteins JGI model ID 239218 and JGI model ID 223016. Further searches of the *C. intestinalis *genome and EST databases available at JGI did not identify other candidate genes for chitinase/chitolectin proteins. Thus, each of the major mammalian phylogenetic groups (Figure 1) must be represented in the *C. intestinalis *genome by a single gene. Three of the *S. purpuratus *proteins [GenBank:XP_798621, XP_783710, and XP_798628] are orthologs of the mammalian chitobiases. The protein XP_783668 is an ortholog of mammalian stablin-1 interacting chitin-binding protein. As described previously for the *D. discoideum *genome, searches of the *S. purpuratus *genome did not identify genes with homology to the mammalian chitinases/chitolectins.

Collectively, the *C. intestinalis *and *S. purpuratus *data, which identify a single chitinase/chitolectin gene in *C. intestinalis *and none in *S. purpuratus*, indicated that a selective expansion of this group likely occurred later in deuterostome evolution to produce the current mammalian chitinase/chitolectin phylogenetic group (Figure 1). The two outlying groups, chitobiases and stablin-1 chitolectins, did not undergo a similar expansion. Notably, the *C. intestinalis *genome assembly v2.0 (March 2005) represents the third assembly release, and is essentially complete with some scaffolds (179 Mb) not yet mapped to chromosomes [50]. The *S. purpuratus *genome sequencing is in progress; therefore, the preliminary assembly (build 1.1) [51] may not include all GH18 family genes.

### Vertebrate Chitinases/Chitolectins (*X. tropicalis, G. gallus, M. domestica*)

Searches of the *X. tropicalis *genome (August 2005 Assembly) and the *G. gallus *genome (May 2006 Assembly) for GH18 family genes indicated that each of these non-mammalian vertebrate genomes includes a single cluster of chitinase homologs as well as one chitobiase and one stablin-1 interacting protein homolog (additional file 5: Other deuterostome sequences). In the case of *X. tropicalis*, a 95 kb region of scaffold 41 encodes three chitinase genes [Entrez Genes 548945, 548404 and 448265], each including a GH18 domain and a CBM14 domain. An 18 kb region of *G. gallus *chromosome 26 encodes two chitinase genes with GH18 and CBM14 domains [Entrez Genes 41993, 395072] and an open reading frame encoding a partial GH18 domain, possibly a pseudogene. The presence of the proton-donating glutamate in the active site of the GH18 domains predicts that the *X. tropicalis *and *G. gallus *gene products are active chitinases. The data showing close clustering of the *X. tropicalis *and *G. gallus *chitinase genes indicate that tandem duplications at a single locus occurred in the evolution of the gene families of these species. A possibility based on observation of conserved synteny is that the *X. tropicalis *scaffold 41 and the *G. gallus *chromosome 26 may represent current versions of an ancestral GH18 locus that expanded and rearranged in mammals after the mammalian-avian split (approx. 310 mya) [52]. The present overall arrangement of the mammalian genes indicates both an expansion and separation of the chitinase/chitolectin genes after the last common ancestor of birds and mammals

*Monodelphis domestica *(the gray short-tailed opossum) was interesting to examine as a representative of an intermediate between the existing mammalian and non-mammalian vertebrate models. As a result of common ancestry, the opossum GH18 family genes may reveal changes that reflect innovations that accompanied mammalian evolution after the avian split. The metatherian ("marsupial") mammals are phylogenetically distinct from current placental mammals having diverged approximately 150–180 mya; however, they are more closely related to one another than to any other vertebrates (birds, amphibians, fishes) [53,54].

The opossum genome includes, on chromosome 2, seven Genescan Gene Prediction models [55] encoding GH18 family proteins (additional file 5: Other deuterostome sequences). Six of the models (c_307.104 through c_307.108 and c_214.85) predict chitinase/chitolectin homologs and the seventh model (c_199.2) predicts a human chitobiase homolog. Three of the gene models (c_307.104, c307.107, and c_214.85) include two GH18 domains. The models encoding chitinase/chitolectins form clusters at the 107 Mb and the 542 Mb regions of chromosome 2, and the chitobiase model is located at 44 Mb. This arrangement resembles that of human GH18 family genes on chromosome 1.

Whole genome alignments of the human genome with the opossum genome (Jan 2006 Assembly) indicated that human genes CHI3L2, RP11-165H20.1, CHIA and OVGP1 align, respectively, with Genescan models c_307.104 (first GH18 domain), c_307.105, c_307.106, and c_307.108. Human genes CHI3L1 and CHIT1 align with the two domains of c_214.85. Thus, the opossum genome includes in the same orientation and order an orthologous gene for each human chitinase/chitolectin, and the human family GH18 family gene arrangement extends at least as far back as marsupials. The opossum genome includes in the same region an additional gene model (c_307.107) with two GH18 domains, which is not included in the human orthology. Further investigation indicated that these two domains (denoted c_307.107A/B in Figure 7, discussed below) are co-orthologs of the *X. tropicalis *putative chitinase [Entrez Gene 448265]. Congruency in number and physical arrangement of GH18 family genes in the opossum and human genomes indicates that an expansion and separation of chitinase/chitolectin genes occurred before the divergence of the metatharian and placental mammals (150–180 mya).

### Chitinase/Chitolectin Encoding Regions Are Adjacent to the Human MHC Paralogous Region at Chromosome 1

**Figure 5:**
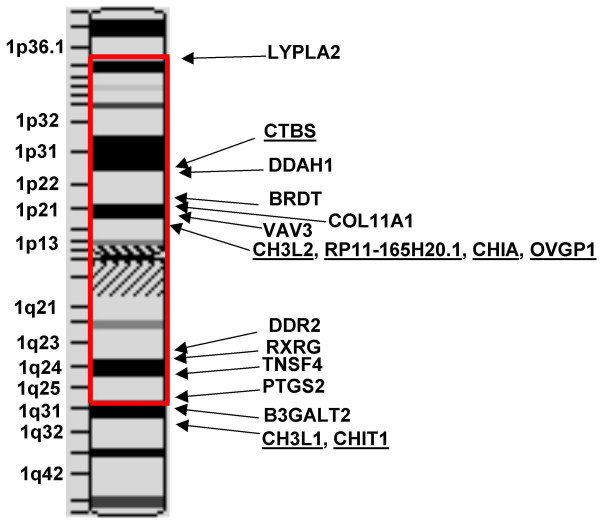
**Proximity of the human family GH18 genes to the human MHC paralogon on chromosome 1**. An ideogram from NCBI Map Viewer shows human chromosome 1 with region encoding MHC paralogon genes boxed in red. Arrows point to the locations of the human family GH18 genes (symbols underlined to distinguish them from MHC related genes) and representative genes from the MHC paralogous region. The choice of genes is based on a recent comparative analysis of the MHC [48]. The MHC paralog symbols are: LYPLA2, lysophospholipase II (1p36.12-p35.1); DDAH1, dimethylarginine dimethylaminohydrolase 1 (1p22); BRDT, bromodomain, testis-specific (1p22.1); COL11A1, collagen, type XI alpha 1 (1p21); VAV3, vav 3 oncogene (1p13.3), DDR2, discoidin domain receptor family, member 2 (1q12-q23); RXRG, retinoid X receptor, gamma, 1q22-q23); TNFS4, tumor necrosis factor (ligand) superfamily, member 4 (1q25); PTGS2, prostaglandin-endoperoxide synthase 2 (1q25.2-q25.3); and B3GALT2, UDP-Gal:beta GlcNAc beta 1,3-galactosyltransferase, polypeptide 2 (1q31).

An interesting observation in view of the recent discovery of an association of the chitinase proteins with Th2 cell mediated immunity (discussed earlier [5,6]) is that the human GH18 family genes are located adjacent to the MHC paralogon genes on the two arms of human chromosome 1 (Figure 5). The human MHC consists of a large number of genes essential to antigen presentation and immune function, including the MHC class I and class II genes that serve pivotal roles in adaptive immunity. In addition to the MHC locus at chromosome 6p21.3, the human genome includes three paralogous genomic regions (paralogons) located on different chromosomes (chromosome1p11-p32/1q21-q25, chromosome 9q32-q34, and chromosome 19 p13.1-p13.3) [48]. The GH18 cluster including CHI3L2, RP11-165H20.1, CHIA, and OVGP1 is located at 1p13.3-p13.2 and the cluster including CHI3L1 and CHIT1 is located at 1q32.1.

**Figure 6:**
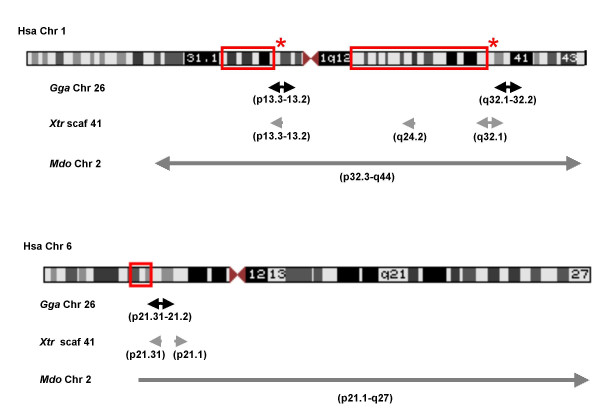
**Syntenic relationships of *X. tropicalis, G. gallus, M. domestica *and human genomic regions involving MHC**. The red rectangles on ideograms of human chromosomes show the locations of the MHC (chr 6) and the two MHC paralogous regions (chr 1). Red asterisks indicate the loci for the human chitinase/chitolectin genes on chr1 (p13.3-p13.2) and chr1 (q32.1). The labeled horizontal arrows below the two ideograms designate the regions and orientations of human chromosomes 1 and 6 that correspondingly align with *G. gallus *chromosome 26 (black); *X. tropicalis *scaffold 41 (gray), and *M. domestica *chromosome 2 (diagonal-pattern).

Furthermore, regions of human chromosomes 1 and 6 adjacent to and including the human MHC, MHC paralogon, and GH18 family genes align with chitinase-containing regions of *G. gallus *chromosome 26, *X. tropicalis *scaffold 41, and *M. domestica *chromosome 2 (Figure 6). Taken as a whole, as much as 82% of the 5.1 Mb *G. gallus *chromosome 26, which encodes the chitinase genes, aligns with regions adjacent to the human MHC and the MHC paralogon at chromosome 1. The *G. gallus *MHC, however, is located separately in a 92 kb region of chromosome 16 (a microchromosome of 433 Kb). This region is generally considered to represent a compact version of the more complex human MHC as it consists of only 19 genes, virtually all having counterparts in the human genome [56]. The *X. tropicalis *scaffold 41 (3.9 Mb) is almost the same size as *G. gallus *chromosome 26 and shows similar synteny with human chromosomes 1 and 6 (Figure 6).

The opossum MHC, which is similar in size and complexity to MHCs of eutherian mammals [57], is located on chromosome 2 (260 Mb region). Opossum chromosome 2 not only contains the GH18 family genes, but also its alignments with the human genome encompassed extensive regions of human chromosomes 1 and 6 including, respectively, the MHC paralogon and the MHC (Figure 6). Approximately 40% of opossum chromosome 2 aligned with the human chromosome 1 and another 40% with human chromosome 6. From the perspective of the human genome, as much as 80% of the 247 Mb human chromosome 1 and 85% of the 170 Mb human chromosome 6 aligned with opossum chromosome 2. Collectively, these data from alignment of the human genome with the *X. tropicalis*, *G. gallus*, and opossum genomes demonstrate conserved general patterns of synteny between family GH18 genes and regions including or adjacent to the MHC and MHC paralogon 1. This conserved synteny suggests an ancient organizational relationship between the MHC and GH18 genes.

### Speculations Regarding the Chitinase/Chitolectin Genes

Host defense associated with innate immunity is one of the important established functions of chitinases, extending as far back in evolution as the common ancestor of plant and animal kingdoms [11]. However, the recent descriptions of the involvement of chitinases/chitolectins in human and mouse T cell effector mechanisms [5,6] suggest an additional role concerned with the adaptive Th2 immune response in mammals. The evolution of an adaptive immune system with somatic rearranging genes is a relatively recent occurrence. Current evidence indicates that genes critical for adaptive immunity first appeared in jawed vertebrates (gnathostomes) about 500 mya [58]. Comparative genomics of the MHC indicate that the paralogous regions on chromosomes 1, 6, 9, and 19 may have originated by block or whole genome duplications from a common ancestral proto-MHC region present in protochordates (*Ciona *and *Branchiostoma*) [48,59,60]. Previous studies indicate that two rounds of whole genome or large block duplication took place before the emergence of jawed vertebrates, but after the emergence of cephalochordates. A recent review discusses the duplications and the architecture of such a proto-MHC in protochordates, and suggests that this region may have included genes that evolved long before the emergence of the adaptive immune system [48]. Immune-related genes may have been among early genes that were co-opted or recruited for additional roles related to adaptive immunity [47,61]. Earlier references [5,6] as well as a recently published review describing chitinases and chitiolectins as "critical mediators" of Th2 inflammatory responses [62] are consistent with the view that chitinase/chitolectin genes were among those recruited.

Considering these new observations related to the evolutionary history of the chitinases, involvement of mammalian GH18 proteins in both innate and adaptive immunity may be related to their expression in phagocytic cells, particularly macrophages [20,21,63] and dendritic cells [64], which have roles in both types of immunity [65]. The association of GH18 proteins with phagocytosis is evident early in animal evolution, as we identified five homologs of the human lysosomal chitobiase in the *D. discoideum *genome. Since *D. discoideum *cells are professional phagocytes that engulf and digest bacteria as a nutrient source, these enzymes are likely among the hydrolases in their phagosomes and lysosomes involved in digestion of bacterial cell walls [25]. During metazoan evolution, the primary digestive function of phagosomes has expanded to include roles in development, tissue remodeling, apoptosis, and in innate and adaptive immunity [66]. In mammalian phagocytic cells, engulfment, and degradation of pathogens as well as mechanisms related to MHC II antigen presentation are associated with endosomal/lysosomal compartments, where several members of the GH18 family are located [20,23,67]. Therefore, the chitinase/chitolectin genes may have evolved and expanded during vertebrate evolution in association with evolutionary changes in phagocytosis, particularly changes associated with vesicular antigen processing pathways.

Many observations are now remarkably consistent with chitinase family GH18 proteins having roles in vesicular antigen processing and MHC II presentation: the requirement of AMCase (CHIA) enzymatic activity for its mediation of Th2 responses in inflammation and asthma [5]; an acidic pH optimum of AMCase catalytic activity corresponding to the acidic endosomal/lysosomal compartments involved in antigen processing [17]; the presence of AMCase (CHIA) and other chitinase/chitolectins, particularly mouse Ym1 (Chi3l3) and human chitinases, in macrophages and other antigen presenting cells [5,63,68,69]; and regulation of both chitinase and chitolectin gene expression by inflammatory cytokines such as interferon γ, IL-4 and IL-13 [5,63,68-70]. An interesting speculation is that chitinase-related genes were present in an ancestral proto-MHC region and were among those recruited for additional roles in immunity. The patterns of conserved synteny of GH18 proteins with the MHC and the chromosome 1 paralogon are consistent with this possibility. Recently the organization of the proto-MHC region of the cephalochordate amphioxus *Branchiostoma floridae *(traditionally considered the closest living invertebrate to the vertebrates, a view recently challenged by new phylogenic data [71]) became available and was used to reconstruct chordate and gnathostome ancestor proto-MHC regions [72]. Interestingly, a homolog for the human NEK7 gene, located at 1q31.3, approx. 5Mb centromeric to the CHIT1 gene, is present in the reconstructed proto-MHC. In *X. tropicalis*, an NEK7 homolog is located on scaffold 41 approx. 4 Mb upstream of the chitinase gene cluster. The opossum chromosome 2 also encodes a NEK7 homolog (located at 99 Mb, 8 Mb upstream of the CHIT1 homolog located at 107 Mb). Thus, synteny between ancient proto-MHC genes and chitinase family members might have existed at least since the emergence of amphibians (approx. 350 mya) [73].

### Evolution by a birth-and-death process

**Figure 7:**
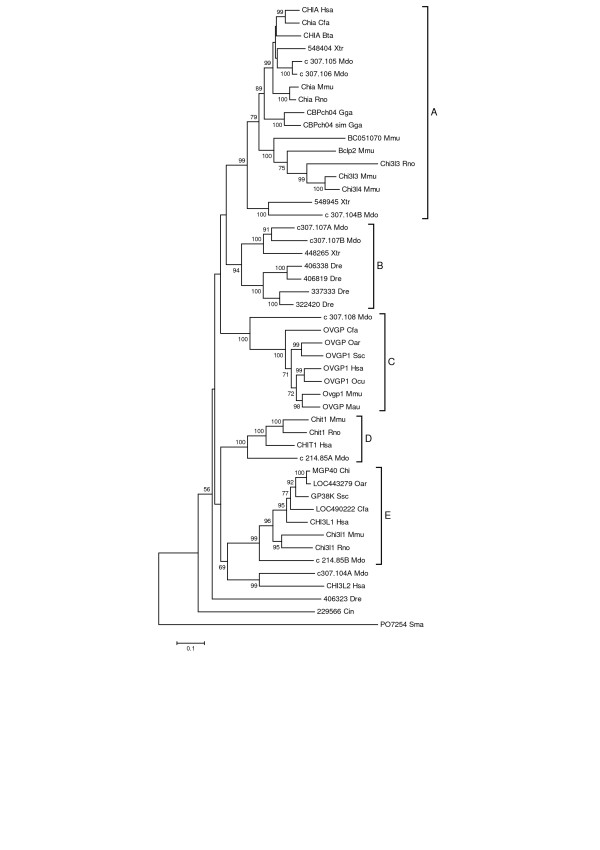
**Phylogenetic tree of chordate chitinases/chitolectins**. The figure shows the minimum evolution tree from a phylogenetic analysis conducted as described in the legend for Figure 1. Besides mammals, the species are *X. tropicalis *(Xtr), *D. rerio *(Dre), *G. gallus *(Gga), *M. domestica *(Mdo) and *C. intestinalis *(Cin). Gene or protein symbols are used for mammals; Gene ID numbers for *X. tropicalis*, *D. rerio *and *G. gallus*; Genscan Gene Prediction symbols for *M. domestica*; and the JGI Model protein ID number for *C. intestinalis*. Groups A-E and their correspondence to subgroups I-IV in Fig. 1 are described in the text. Scale, rooting and bootstrapping are as described for Figure 1. Bootstrap values ≥ 50% are shown.

Our comparative genomic analysis of the GH18 protein family indicates that considerable loss, duplication and diversification of these genes occurred in vertebrates. The diversification and expansion occurred in the chitinase/chitolectin phylogenetic group with no indication of similar changes in the chitobiases and stabilin-1 interacting groups. A phylogenic tree, Figure 7, shows the relationship of homologous chitinase/chitolectin genes from several vertebrate species including six chitinase genes from *D. rerio *(zebrafish), which shared a common ancestor with mammals approximately 420 mya [53]. The tree also includes the *C. intestinalis *chitinase [JGI protein model ID 229566] and is rooted with the bacterial GH18 protein *Serratia marcescens *[GenBank:P07254]. As observed for earlier metazoan species, Figure 7 shows that distantly related vertebrates such as humans, *M. domestica, G. gallus, X. tropicalis*, and *D. rerio *have significantly different sets of chitinase/chitolectin genes. According to the tree, the genes form four major clusters (A, B, C, D). Cluster A corresponds to the chitinase/chitolectin subgroup I (Figure 1); cluster C, to subgroup IV; cluster D, to subgroup II; and cluster E to subgroup III. Cluster B does not include genes from placental mammals and is not included in Figure 1. The dynamic evolutionary process for these genes must reflect the morphological and other changes that accompanied the stages of vertebrate evolution.

The process of gene expansion, deletion and diversification must have occurred more than once to explain the long evolutionary history of the GH18 family of chitinase genes. Although the literature indicates multiple chitinases are present in plants and bacteria, we clearly identified chitobiase genes, but no chitinase/chitolectin genes in the nearly complete *D. discoideum *genome. An important expansion of chitinase/chitolectin genes must have occurred in protostomes *C. elegans *and *D. melanogaster*, as chitinous structures in these species became important protective and architectural features. Chitin synthase (biosynthesis) and chitinases lost importance in early deuterostomes, and examination of chitinase/chitolectin genes in *C. intestinalis *and *S. purpuratus *genomes indicated that the expansion and diversification of chitinases/chitolectins did not continue in these early non-chitinous deuterostomes (Figure 4). However, comparison of the genes from these early genomes with higher vertebrate and mammalian genomes indicated a significant expansion of genes during late vertebrate evolution. Comparison of GH18 family genes from *X. tropicalis*, *G. gallus *and *M. domestica *revealed an increase of chitinase/chitolectin genes after the avian-mammalian split (Figure 7).

**Figure 8:**
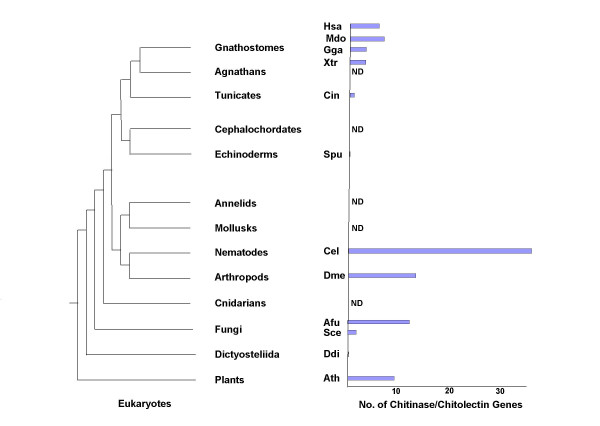
**Summary diagram indicating contraction and expansion of the chitinase/chitolectin protein family during eukaryotic evolution**. A schematic of eukaryote phylogeny is shown alongside a bar graph indicating the number of chitinase/chitolectin genes identified in the genomes of selected species corresponding to the tree classifications. The schematic is according to the recent animal phylogeny suggested by multigene analyses of bilaterian animals [71]. The species for which the number of genes is shown are the gnathostomes (jawed-vertebrates) human (Hsa), opossum (Mdo), chicken (Gga) and Xenopus (Xtr); the chordate *C. intestinalis *(Cin); the echinoderm *S. purpuratus *(Spu); the nematode *C. elegans *(Cel); the arthropod *D. melanogaster *(Dme); and the Dictyosteliida *D. discoideum *(Ddi), all discussed in the text. Also included in the graph are data for the fungi *Aspergillis fumigatus *and *Saccharomyces cerevisiae *and the plant *Arabidopis thaliana *(Additional file 7: Other chitinase sequences). The *A. fumigatus *and *S. cerevisiae *genes were obtained by querying Fungal Genomes Central at NCBI.

Figure 8 provides a summary of the contraction and expansion of these genes during eukaryotic evolution. Overall, the data presented show a general lack of orthology between the protostome, early deuterostome, and mammalian chitinases/chitolectins and support the view that the mammalian genes evolved new functions associated with innovations that accompanied evolution of the mammalian species. The phylogenetic data for the GH18 family are consistent with the model of birth-and-death evolution described by Nei and colleagues [74].

The evolutionary history of the GH18 family includes other examples of recruitment of family members for novel functions. The emergence of a subfamily of xylanase inhibitors within the GH18 family recently reported in plants represents one such example [75]. A factor central to the evolution of novel functions for the GH18 family may be their very common protein fold, the TIM barrel conformation, known to form a stable core structure with specific binding and catalytic functions residing in the loop regions separated from the alpha helices and beta sheets. This type of structure/function relationship in proteins may be particularly well suited to divergence of new functions from an ancestral protein by the process of gene duplication and subsequent modification by mutation, since changes in the loop regions would not produce major changes in protein stability [75,76]. McMahon et al. (2005) recently emphasized the importance of scaffold stability in the evolution of proteins displaying sequence variation related to binding diversity [77]. To our knowledge, recruitment for development or host defense purposes has not been reported for other GH families.

## Conclusion

Our comprehensive genomic study of animal GH18 family proteins provides valuable new insights into phylogenetic relationships of the proteins and the evolutionary history of the protein family. This represents the first such detailed study of animal GH18 family proteins. The family consists of three major phylogenetic groups: chitobiases, chitinases/chitolectins, and stabilin-1 interacting chitolectins. Only the chitinase/chitolectin group is associated with expansion in late deuterostomes. The phylogeny of animal GH18 genes is consistent with evolution of the family by a birth-and-death process according to the model described for multigene families of the vertebrate immune system [74]. The finding that the human GH18 gene family is closely associated with the human MHC paralogon on chromosome 1, together with published data indicating an association of GH18 chitinase activity with Th2 cell inflammation, suggests the possibility that the late expansion could be related to an emerging interface of innate and adaptive immunity during early vertebrate history.

## Methods

### Genome searches

For *C. elegans *and *D. melanogaster *genomes, BLASTP was used to query the NCBI Entrez Genome nucleotide and protein databases with the complete human chitinase sequences [GenBank:NP_970615 and NP_003456] and the GH18 domain sequences. The Entrez protein link to the NCBI's Conserved Domain Database search service (CDD) [78] identified the GH18 domain family members (GH18 is designated CDD|24313 which corresponds to pfam00704, smart60636, and Interpro IPR001223 in the respective domain-based databases). The BLAST protein searches used the Entrez protein graphical viewer BLink [79] to identify closely related sequence neighbors. Other resources were Ensembl v37 – February 2006 [80] used to search for IPR001223 domains, Flybase [36] and Aceview [35].

Query of *Ciona intestinalis *genome sequence v2.0 (March 2005) available from JGI Joint Genome Institute, (JGI) [49] using human protein sequences and GH18 domains [GenBank:NP_970615 and NP_003456] along with searches of the *C. intestinalis *genome using the domain GH18 [Interpro IPR001223] identified the *C. intestinalis *chitinase-related genes.

The human chromosome 1 regions encoding chitinase/chitolectin genes were compared to whole genome assemblies from *Monodelphis domestica*, *Gallus gallus*, *Xenopus tropicalis *and *Danio rerio *using the UCSC browser [81]. The UCSC Genome Browser databases [82] used in this study were: NCBI Build 35, May 2004 Assembly (hg17) produced by the International Human Genome Sequencing Consortium; Opossum Jan 2006 (UCSC version monDom4) (produced by The Broad Institute, Cambridge, MA, USA; the February 2004 chicken (*Gallus gallus*) draft assembly, produced by the Genome Sequencing Center at the Washington University School of Medicine in St. Louis, Mo. USA; the August 2005 Assembly frog (*Xenopus tropicalis*) whole genome shotgun (WGS) assembly version 3.0, DOE Joint Genome Institute (JGI) Walnut Creek, CA, USA; the May 2005 zebrafish (*Danio rerio*) Zv5 assembly produced by The Wellcome Trust Sanger Institute in collaboration with the Max Planck Institute for Developmental Biology in Tûbingen, Germany, and the Netherlands Institute for Developmental Biology (Hubrecht Laboratory), Utrecht, The Netherlands.

Other genome resources were: NCBI Entrez genome databases for *Strongylocentrotus purpuratus *(sea urchin) Build 1.1, August 2005; *Dictyostelium discoideum *Build 1.1, November 2005, and *Danio rerio *(zebrafish) Build Zv4, July 2005 and Ensembl genomes *Danio rerio *Zv5, May 2005 Assembly; *Xenopus tropicalis *JGI 4.0 June 2005 Assembly, and *Monodelphis domestica *MonDom 4.0, January 2006 Assembly. In some cases, NCBI Map Viewer [83] was used for chromosomal locations.

### Phylogenetic Analysis

For multiple sequence alignments, the Clustal X (version 1.83) Program [84] aligned the GH18 domain amino acid sequences. Boundaries of GH18 domains were obtained from the multiple sequence alignment by editing multiple alignments to conform to the GH 18 domain of human chitotriosidase (CHIT1), for which the 3D structure is available [PDB: 1HKK]. The alignment parameters used were those suggested by Hall [85]. Values used for pairwise alignments were gap opening penalty 35 and gap extension penalty 0.75. Values for multiple alignment were gap opening penalty 15, gap extension penalty 0.3, and delay divergent sequences 25%. Protein weight matrix chosen from the multiple alignment parameters menu was Gonnet series. Duplicate and alternatively spliced protein isoforms were excluded so that only one gene product was used for analysis. Phylogenetic analyses were conducted with MEGA version 3.1 software [86] using maximum parsimony, minimum evolution, and neighbor-joining methods with the Poisson-correction for multiple amino acid substitutions. In all cases, the three methods produced very similar topologies. A summary composite tree that includes the early eukaryote, protostome, early deuterostome and vertebrate sequences discussed in the text is included as additional file 8: Composite tree.

## Authors' contributions

JDF & NNA conceived the study. JDF collected and analyzed the data and wrote the paper. NNA provided substantial editorial advice and assistance and provided critical insights into GH18 family biochemistry and biology. Both authors have read and approved the final manuscript.

## Supplementary Material

Additional file 1 GH18 Family Domain Structure. Click here for file

Additional file 2 Mammalian Sequences. Click here for file

Additional file 3 *C. elegans *Sequences. Click here for file

Additional file 4 *D. melanogaster *Sequences. Click here for file

Additional file 5 Other Deuterostome Sequences. Click here for file

Additional file 6 Supplementary Phylogenetic Tree. Click here for file

Additional file 7 Other Chitinase Sequences. Click here for file

Additional file 8 Composite tree. Click here for file
