# Supplementary material for: Chitinase family GH18: evolutionary insights from the genomic history of a diverse protein family
Source: BMC Evol Biol. 2007 Jun 26;7:96. doi: 10.1186/1471-2148-7-96 (PMC1945033; doi:10.1186/1471-2148-7-96)
Supplement: Additional file 1 — GH18 Family Domain Structure. [file 1471-2148-7-96-S1.doc]

Chitinase: Glu

Chitolectin: Leu, Ile, Gln

GH18 (β/α)_8_ Barrel: Catalytic Domain

CBM14 Domain(s)

Chitinase GH18 family domain structure― Glycoside hydrolase family 18 consists of both active enzymes (chitinases) and non-active proteins (chitolectins). Both sets of GH18 family proteins possess a main domain having a (β/α)_8_ TIM barrel fold, but the chitolectin group does not hydrolyze chitin because an active-site glutamic acid required to donate a proton during hydrolysis is changed to a leucine, isoleucine or glutamine. Some family members have additional domains, including one or two chitin-binding domains CBM14.
