# Supplementary material for: Chitinase family GH18: evolutionary insights from the genomic history of a diverse protein family
Source: BMC Evol Biol. 2007 Jun 26;7:96. doi: 10.1186/1471-2148-7-96 (PMC1945033; doi:10.1186/1471-2148-7-96)
Supplement: Additional file 6 — Supplementary Phylogenetic Tree. [file 1471-2148-7-96-S6.doc]

Human, *C. elegans*, and *D. melanogaster* minimum evolution tree with *H. echinata* CAG25409 sequence. The bracket indicates the clade discussed in the text. The tree is rooted with *S. marcescens* P07254. Bootstrap values ≥ 70% are shown.
