# Supplementary material for: Chitinase family GH18: evolutionary insights from the genomic history of a diverse protein family
Source: BMC Evol Biol. 2007 Jun 26;7:96. doi: 10.1186/1471-2148-7-96 (PMC1945033; doi:10.1186/1471-2148-7-96)
Supplement: Additional file 8 — Composite tree. [file 1471-2148-7-96-S8.doc]

Composite minimum evolution tree of early eukaryote, protostome, early deuterostome, and vertebrate sequences discussed in the text. The clade indicated in green represents the chitinases/chitolectins (bootstrap value 71%); the clade in olive represents the imaginal disk factors (bootstrap value 100%); the clade in maroon represents chitobiases (bootstrap 99%), and the clade in teal the stablin-1 interacting proteins (bootstrap 95%). A clade consisting of *C. elegans* sequences is collapsed to increase clarity of presentation. The tree is rooted with the *S. marcesens* sequence P07254.
