# Supplementary material for: Chitinase family GH18: evolutionary insights from the genomic history of a diverse protein family
Source: BMC Evol Biol. 2007 Jun 26;7:96. doi: 10.1186/1471-2148-7-96 (PMC1945033; doi:10.1186/1471-2148-7-96)
Supplement: Supplementary file 4 [file 1471-2148-7-96-S2.doc]

	Additional File 2: Mammalian Sequences	
	**Gene or Protein**[1]	**Description (aliases)**
1	CHIT1_Hsa	chitotriosidase
2	CHIA_Hsa	acidic chitinase (AMcase, TSA1902)
3	CHI3L2_Hsa	chitinase 3-like protein 2 precursor (YKL-39)
4	CHI3L1_Hsa	chitinase 3-like 1, cartilage glycoprotein-39 (GP39, HC-gp39, HCGP-3P, YKL40)
5	OVGP1_Hsa	oviduct glycoprotein
6	CHID1_Hsa	chitinase domain containing
7	CTBS_Hsa	chitobiase, di-N-acetyl-
8	Chit1_Mmu	chitotriosidase
9	Chia_Mmu	acidic chitinase (AMCase, YNL)
10	BC051070_Mmu	Hypothetical protein NP_848499.
11	Chi3l3_Mmu	chitinase 3-like 3 (ECF-L, Ym1)
12	Ch3l4_Mmu	chitinase 3-like 4 (Ym2)
13	Chi3l1_Mmu	chitinase3 like 1 (Brp39, Gp39, brp-39)
14	Bclp2_Mmu	chitinase like protein 2
15	Ovgp1_Mmu	oviduct glycoprotein
16	Ctbs_Mmu	chitobiase, di-N-acetyl-
17	Chit1_Rno	predicted chitotriosidase
18	Chia_Rno	chitinase, acidic
19	Chi3l3_Rno	chitinase 3 like 4 predicted
20	Chi3l1_Rno	chitinase 3 like 4 ((cartilage glycoprotein-39)
21	Ctbs_Rno	chitobiase, di-N-acetyl-
22	Chid1_Rno	similar to cc1-9
23	OVGP_Ocu (AAK52727)	development promoting factor precursor
24	OVGP1_Mau (Q60557)	oviduct glycoprotein
25	LOC504781_Bta	MGC3234, MGC140688
26	CHIA_Bta	acidic chitinase
27	LOC479904_Cfa	similar to acidic chitinase (AMCase)
28	LOC609807_Cfa	**similar to [illegible]**
29	CTBS_Cfa	**similar to chitobiase**
30	LOC475993_Cfa	**similar to [illegible]**
31	LOC490222_Cfa	**similar to chitinase 3-like 1**
32	LOC443279_Oar	signal processing protein
33	OVGP_Oar	oviduct protein
34	MGP40_Chi	BP40 precursor
35	GP38K_Ssc	38 kDa heparin-binding glycoprotein
36	OVGP1_Ssc	oviduct glycoprotein

1 Gene symbols (or protein symbols where gene symbols are not available) and binomen abbreviations of the form "Gsp" (Genus, species) are used.
