# Supplementary material for: Chitinase family GH18: evolutionary insights from the genomic history of a diverse protein family
Source: BMC Evol Biol. 2007 Jun 26;7:96. doi: 10.1186/1471-2148-7-96 (PMC1945033; doi:10.1186/1471-2148-7-96)
Supplement: Supplementary file 5 [file 1471-2148-7-96-S3.doc]

	Additional file 3: C. *elegans* Sequences	
	**Gene ID**	**Ref Seq**
1	3565897	NP496030
2	187738	NP496031
3	187736	NP496026
4	187737	NP496027
5	187731	NP496021
6	182431	NP496133
7	174500	NP496018
8	187732	NP496020
9	187733	NP496019
10	187734	NP496023
11	187735	NP496024
12	174502	NP496029
13	174888	NP871952 NP496660
14	182434	NP496125
15	182435	NP496127
16	182436	NP496126
17	182432	NP496128
18	182437	NP496131
19	174541	NP496132
20	174363	NP495798
21	188615	NP496034
22	188616	NP496035
23	188614	NP496033
24	191470	NP496122
25	187741	NP496028
26	187739	NP496032
27	173661	NP494455
28	189507	NP497437
29	177464	NP501082
30	183470	NP500856
31	183471	NP500855
32	187357	NP506770
33	179121	NP504862
34	187942	NP504806
35	187168	NP506735
36	180628	NP508588
37	180447	NP508185
