# Supplementary material for: Chitinase family GH18: evolutionary insights from the genomic history of a diverse protein family
Source: BMC Evol Biol. 2007 Jun 26;7:96. doi: 10.1186/1471-2148-7-96 (PMC1945033; doi:10.1186/1471-2148-7-96)
Supplement: Supplementary file 6 [file 1471-2148-7-96-S4.doc]

	Additional file 4: *D. melanogaster Sequences*			
	**Gene ID**	**Ref Seq**	**Other Designations**	**Chromosome**
1	49815	NP_524962	CG3986-PA;Cht4	2R
2	37390	NP_611542	CG9357-PA	2R
3	37392	NP_611543	CG10531-PA	2R
4	246535	NP_726022	CG30293-PA	2R
5	36868	NP_477081	CG5210-PA, Chit	2R
6	37104	NP_611321	CG5154-PA, Idgf5	2R
7	34981	NP_723967<br>NP_477256<br>NP_723966	CG4559-PA, Idgf3	2L
8	34979	NP_477257	CG4475-PA, Idgf2	2L
9	34978	NP_477258	CG4472-PA, Idgf1	2L
10	41687	NP_650314	CG9307-PA	3R
11	38370	NP_647768	CG1869-PA	3L
12	38223	NP_477299	CG2054-PA, Cht2	3L
13	31926	NP_727374	CG1780-PA, Idgf4	X
14	31630	NP_572361	CG3044-PA	X
15	31935	NP_572598	CG2989-PA	X
16	3355116	NP_001015410	CG18140-PA, Cht 3	Cytogenetic: 40D-40F
17	34115	NP_609190	CG8460-PA (CHID1 homolog)	2L
