# Supplementary material for: Chitinase family GH18: evolutionary insights from the genomic history of a diverse protein family
Source: BMC Evol Biol. 2007 Jun 26;7:96. doi: 10.1186/1471-2148-7-96 (PMC1945033; doi:10.1186/1471-2148-7-96)
Supplement: Supplementary file 7 [file 1471-2148-7-96-S5.doc]

Additional file 5: Other deuterostome sequences		
***Monodelphis domestica* Sequences**		
**Gene or Gene Model Identifer**	**Protein Identifier**	**Description**
c_307.104	none	Genscan Gene Prediction
c_307.105	none	Genscan Gene Prediction
c_307.106	none	Genscan Gene Prediction
c_307.107	none	Genscan Gene Prediction
c_307.108	none	Genscan Gene Prediction
c_214.85	none	Genscan Gene Prediction
c_199.2	none	Genscan Gene Prediction
***Gallus gallus* Sequences**		
ID 419931	XP_418051	Similar to CBPch04
ID 395072	NP_989760	CHIA, CBPch04
Model Chr26_1.223	cDNA: CR406345	Predicted from cDNA
ID424535	XP_422372	chitobiase, di-N-acetyl
ID423103	XP_421037	CHID1
***Xenopus tropicalis* Sequences**		
ID 448265 (JGI Gene 66717)	NP_001005792	cht1-provisional
ID 548945 (JGI Gene 18800)	NP_001016191	hypothetical protein
ID 548404 (JGI Gene 66715)	AAH90382	acidic chitinase
ID493467	NP_00100815	(similar to human CHID1)
ID 497004	NP_001011500	LOC497004 (similar to CTBS)
Danio rerio (zebrafish) Sequences		
ID 406338 (zgc_55941)	NP_998414	LOC406338
ID 406819 (zgc:55406)	NP_998378	LOC406819
ID 406323 (zgc:56053)	NP_998215	LOC406322
ID 337333 (zgc:63792)	NP_956740	chitinase
ID 322420 (zgc:65788)	NP_955897	LOC322420
***Ciona intestinalis* Sequences**		
JGI: cioin2:229566	JGI ID 229566	JGI Gene Model
JGI: cioin2:239218	JGI ID 239218	JGI Gene Model
JGI: cioin2:223016	JGI ID 223016	JGI Gene Model
***Strongylocentrotus purpuratus* Sequences**		
ID 594077	XP_798621	similar to Di-N-acetylchitobiase
ID 578451	XP_783710	similar to Di-N-acetylchitobiase
ID 594065	XP_798628	similar to Di-N-acetylchitobiase
ID 578405	XP_783668	aligns with human CHID1
Hydractinia echinata		
Not available	CAG25409	chitinase 1 precursor
Not available	CAL36995	chitinase 2
Dictyostelium discoideum		
ID 3393954	EAL6889	
ID 3395463	EAL7009	
ID 3394351	EAL69263	
ID 3388787	EAL63697	
ID 3382788	EAL63698	
