# Supplementary material for: Chitinase family GH18: evolutionary insights from the genomic history of a diverse protein family
Source: BMC Evol Biol. 2007 Jun 26;7:96. doi: 10.1186/1471-2148-7-96 (PMC1945033; doi:10.1186/1471-2148-7-96)
Supplement: Supplementary file 8 [file 1471-2148-7-96-S7.doc]

Additional file 7: Other Chitinase Sequences

Aspergillus fumigatus (filamentous fungus)		
Gene ID	Gene Designation	Protein Identifier
3508109	Afu6g 09310	XP_750818
3512520	Afu3g11280	XP_754491
3504437	Afu8g01410	XP_747065
3505758	Afu5g01400	XP_748202
3506269	Afu7g08490	XP_748766
3508172	Afu6g09780	XP_750867
3505619	Afu5g03960	XP_747948
3507821	Afu1g02800	XP_750029
3508563	Afu613720	XP_751248
3510929	Afu506840	XP_753949
3511956	Afu3g07160	XP_754890
3507527	Afu7g00310	XP_749783
Arabidopsis thaliana (mouse-ear cress)		
827716	AT4G19720	NP_193707
827717	AT4G19730	NP_193708
827718	AT4G19740	NP_193709
827719	AT4G119750	NP_193710
827720	AT4G119760	NP_193711
827721	AT4G119770	NP_193712
827724	AT4G119800	NP_193715
827725	AT4G19810	NP_193716
827726	AT4G19820	NP_193717
Saccharomyces cerevisiae (yeast)		
851977	YDR371W	NP_010659
850992	CTS1; YLR286C	NP_013388
